# Expression of the Fatty Acid Receptor GPR120 in the Gut of Diet-Induced-Obese Rats and Its Role in GLP-1 Secretion

**DOI:** 10.1371/journal.pone.0088227

**Published:** 2014-02-10

**Authors:** Sarah Juel Paulsen, Leif Kongskov Larsen, Gitte Hansen, Shekar Chelur, Philip Just Larsen, Niels Vrang

**Affiliations:** 1 Department of Histology, Gubra, Hørsholm, Denmark; 2 Department of Molecular Biology, Vipergen Aps, Copenhagen, Denmark; 3 Department of Preclinical Biology, Aurigene Discovery Technologies Ltd, Bengaluru, India; 4 Department of Diabetes Research and Translational Science, Sanofi-Aventis, Frankfurt Am Main, Germany; GDC, Germany

## Abstract

Stimulation of the G protein coupled receptor GPR120 has been shown to have anti-inflammatory and insulin-sensitizing effects, to promote glucagon like peptide-1 (GLP-1) secretion, and to play a key role in sensing dietary fat and control energy balance. In a search for differentially expressed genes potentially involved in food intake and body-weight regulation we identified GPR120 to be differentially regulated in the intestine of selectively bred diet induced obese (DIO) and diet resistant (DR) rats. Subsequently we investigated the effect of GPR120 receptor stimulation with the long chain fatty acid alpha linolenic acid (ALA) on GLP-1 secretion in rats. Independent of diet (high or low fat), GPR120 expression showed a two-fold increase in the intestine of DIO compared to DR rats. In situ hybridization revealed a broad expression of GPR120 in the gut mucosa in both intestinal epithelial and endocrine cells. Using double in situ hybridization GPR120 mRNA did not appear to be enriched in preproglucagon expressing L-cells. In line with the anatomical data, ALA administration did not increase circulating GLP-1 levels. Our data shows a widespread expression of GPR120 in the gut epithelium and can not confirm a major role for GPR120 in the regulation of GLP-1 secretion. The broad expression of GPR120 in the gut epithelium supports reports indicating a putative role of GPR120 as a sensor of dietary fat.

## Introduction

GPR120 belongs to a class of five G-protein coupled receptors (GPR40, GPR41, GPR43, GPR84 and GPR120) that are activated by free fatty acids (FFAs). GPR41 and GPR43 are engaged by short chain fatty acids, GPR84 by medium chain and GPR40 and GPR120 by long chain fatty acids [1], [2], [3], [4]. GPR120 was initially demonstrated to be the functional receptor for the essential fatty acid omega-3-fatty acid (ω-3-FA) alpha-linolenic acid (ALA) [1]. It was shown to be expressed on endocrine L-cells lining the gut and to directly mediate ALA-induced increases in GLP-1 [1], pointing to GPR120 as a potential diabetes target. Recently, Oh *et al* [5] demonstrated that GPR120 serves as a sensor for ω-3-FA. In mice they found GPR120 highly expressed in adipose tissue and CD11c^+^ pro-inflammatory macrophages, and cellular assays showed that activation of the receptor was anti-inflammatory (inhibited pro-inflammatory pathways activated by for example TNF-alfa [5]). Additionally, they showed that the anti-inflammatory signalling properties of GPR120 were involved in the insulin sensitizing and anti-diabetic effects of ω-3-FA [5]. Data further supporting a key role of GPR120 in metabolism come from a recent publication demonstrating a role for GPR120 in obesity [6]. Thus, compared to wild type mice, GPR120 KO mice were shown to be more obesity prone on a high-fat diet, to show decreased adipocyte differentiation, fatty liver, glucose intolerance and insulin resistance – all together indicating that GPR120 is an important lipid sensor involved in fat cell differentiation and body-weight regulation [6].

In a population study, Ichimura *et al* subsequently showed up-regulation of GPR120 in omental fat from obese humans, identified a non-synonymous mutation in the GPR120 receptor in humans (p.R270H) that inhibits GPR120 signalling activity, and showed that pR270H was significantly associated with morbid obesity in European populations [6]. The effect on body mass of genetic variations in the GPR120 gene has also been examined in a Japanese population [7]. Here a possible link between body mass index and common variations in the GPR120 gene and dietary fat intake was observed.

Interestingly, in a search for differentially regulated genes using microarray analysis we identified GPR120 mRNA to be upregulated in gut extracts from diet-induced obese rats when compared to diet-induced resistant rats (Paulsen *et al*, unpublished observations). Given the initial observations of GPR120 as a receptor for ALA and its location on gastrointestinal endocrine cells we aimed to shed further light on the role of this receptor in the gut. First we aimed to verify the differential expression of GPR120 in DIO vs. DR rats, next we aimed to anatomically locate the receptor in the gut using radioactive in situ hybridization and finally we wanted to investigate the role of the GPR120 receptor on the secretion of GLP-1 from the intestinal L-cells lining the gut.

## Materials and Methods

### Animals

All experiments were conducted in accordance with internationally accepted principles for the care and use of laboratory animals and were approved by the Danish committee for animal research. A total of 48 male diet induced obese- (DIO), 48 male diet resistant- (DR) (Rheoscience breeding colony; Rheoscience, Denmark), 24 male Wistar- and 26 male Sprague-Dawley- (SPD) rats (Charles River, Germany) were used. All animals were kept on a 12-h light, 12-h dark cycle (lights on at 6 AM) in a temperature-controlled environment (22–24°C) with free access to food and water unless otherwise stated.

The original breeding colony of DIO and DR rats was developed from a SPD background [8]. On the day of weaning DIO and DR rats were housed individually and stratified according to body weight into two groups offered either chow diet (Altromin standard #1324 chow; C. Petersen, Ringsted, Denmark) or energy-dense high-fat diet (HE; 4.41 kcal/g - Energy %: Carbohydrate 51.4 kcal %, Fat 31.8 kcal %, Protein 16.8 kcal %; diet #12266B; Research Diets, New Jersey, USA) and water. The Wistar and SPD rats were fed the standard chow diet.

### Determination of gene expression levels in colon and ileum

DIO and DR rats fed chow- or HE-diet were terminated at 6 and 12 months of age (n = 8). Two segments of colon (2 cm) and ileum (2 cm) adjacent to the appendix were isolated, rinsed, and immediately homogenised in TRIzol® reagent (Life technologies™). RNA was isolated as described by the manufacturer. Quantitative PCR was performed as previously described [9]. Briefly, first-strand cDNA was prepared in a 20μl reaction using 1 µg total RNA, the Superscript II RT kit (Life technologies™), and random hexamer primers. The cDNA was diluted 1:6 in water and a PCR mixture was prepared. For 13.5 µl: 1.35 µl 10x polymerase buffer, 0.20 µl dNTP (4 mM, 2 mM dCTP), 0.25 µl of each primer (10 µM), 0.125 µl Taq polymerase, 0.0625 µl ^33^P-α-dCTP (10 mCi/ml) (Amersham Pharmacia), 1.5 µl cDNA solution, and finally water to 13.5 µl. Two primer sets were included in each reaction, one set specific for rat GPR120, the second set specific for rat TATA-box binding protein (TBP). The primers were: GPR120: 5′-GACCAGGAAATTCCGATTTG-3′ and 5′-CTGGTGGCTCTCGGAGTATG-3′. TBP: 5′-ACCCTTCACCAATGACTCCTATG-3′ and 5′-TGACTGCAGCAAATCGCTTGG-3′. All samples were subjected to 25 cycles of amplification by PCR. The final PCR reactions were separated on a 6% (w/v) polyacrylamide gel, containing 7 M urea. The gel was subsequently dried, exposed to a phosphorimager screen, and the resulting image was analyzed. Finally, the GPR120 expression was normalized to the TBP expression.

### In situ hybridisation (ISH)

Intestines from 2 male SPD rats were removed and snap frozen on dry ice. Single and double ISH using riboprobes was performed as previously described [10]. RNA probes were directed against rat GRP120 and rat preproglucagon. Sense RNA probes were used as negative controls. The GPR120 probe was labelled radioactively by the use of ^33^P-αUTP (Amersham Pharmacia), and the preproglucagon probe was labelled by use of digoxigenin-11-UTP (Roche Applied Science).

### Oral linolenic acid tolerance test (OLTT)

The rats were housed singly, handled daily, and subjected to mock gavages with water for at least 6 days before the test was performed. 3 month old DIO/DR rats were randomized into 4 active-GLP-1 concentration-matched groups (n  =  8) for alpha-linolenic acid/octanoic acid gavage. Sprague-Dawley and Wistar rats were randomised by weight (n = 8). ALA and octanoic acid (Sigma-Aldrich) were diluted in 5% PEG200 (Sigma-Aldrich) and dosed as 28 mg/kg. Glucose (Frescenius Kabi, Sweden) in a concentration of 500 mg/L was administered as 2 g/kg. For the OLTT all animals were mildly fasted as they received 50% of their normal food consumption the day before the experiment. The compounds were administered as a gavage via a gastrically placed tube connected to a syringe ensuring accurate dosing. Blood samples were obtained from the tail vein at time points 0 (before dosing), 15, 30, 60 and 120 min (DIO-DR), 0, 15, 60, 90 and 120 min (SPD) or 0, 30 and 60 min (Wistar) after dosing, DPP4 inhibitor (Millipore) was added and plasma isolated for the measurement of hormone levels.

### Hormone measurements

Active GLP-1 and insulin levels were determined using Lincoplex (Millipore) or ELISA kits (Millipore) as described by the manufacturer.

### Statistical analysis

Results are presented as mean ± standard error of the mean. Two-way ANOVA with Bonferroni’s multiple comparisons correction (repeated measures) was used for analysis of OLTT and gene expression data. One way ANOVA followed by Fisheŕs post hoc test was used to test for difference between groups. Differences were considered statistically significant when *P* < 0.05. All statistical calculations and relevant graphs were prepared using GraphPad Prism. Images were adjusted for brightness and contrast in Adobe Photoshop and assembled into plates using Adobe Illustrator (CS5).

## Results

### Multiplex PCR analysis

**Figure 1:**
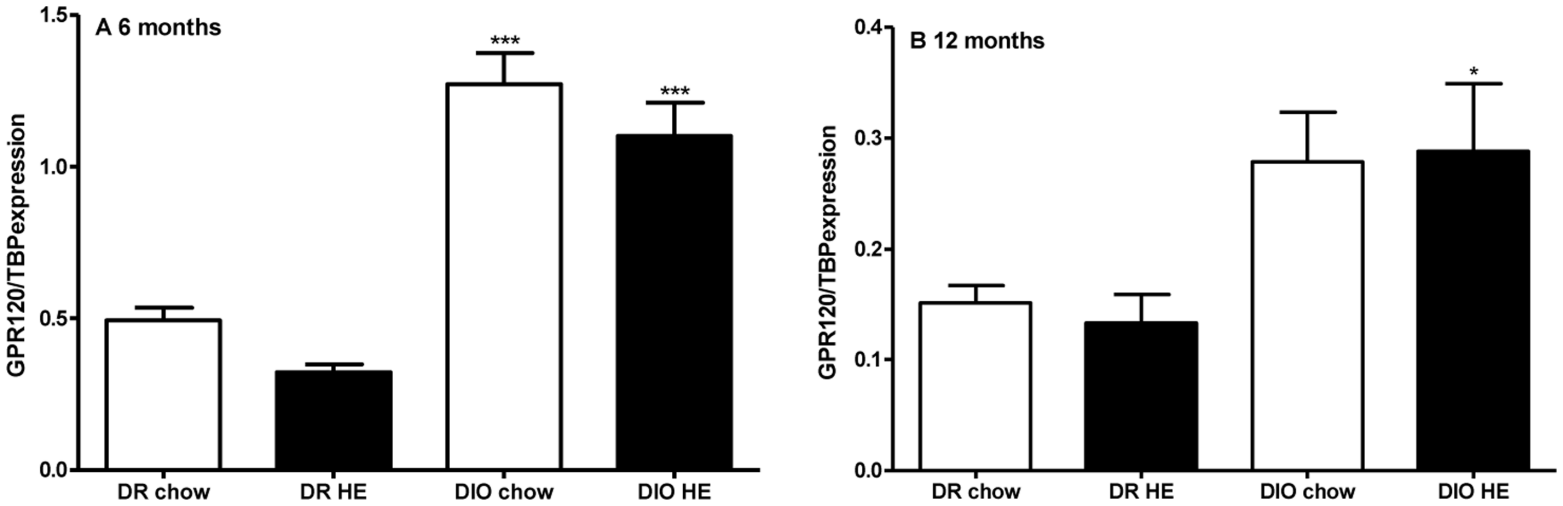
Expression of GPR120 in the intestine of DR and DIO rats aged 6 (a) and 12 (b) months. An L-cell rich part of the intestine (distal ileum) was used for RNA isolation and subsequent quantification by PCR. Two way ANOVA revealed a main effect of both genotype at 6 and 12 months of age (F = 96.15, P<0.001 and F = 14.42, p = <0.001 respectively). There was a main effect of diet at 6 but not 12 months of age (F = 4.69, p = 0.04 and F = 0.16, p = 0.69 respectively). There was no interaction between genotype and diet at 6 or 12 months. One way ANOVA showed statistical differences between both chow and HE fed DR and DIO rats at 6 months of age (p<0.001), and chow and HE fed DIO rats at 12 months of age (p<0,05). * = p<0.05; *** = <0.001.

The expression of GPR120 was examined in an L-cell rich part of the intestine (terminal ileum and proximal colon) of DIO and DR rats fed either chow- or HE diet from weaning to 6 and 12 months of age respectively. Two-way ANOVA revealed a main effect of genotype at both time-points (6 months F = 96.15, P<0.001; 12 months F = 14.42, p = <0.001), with DIO rats consistently showing the highest level of GPR120 expression (fig. 1). At six months of age a main effect of diet was observable (6 months F = 4.69, p = 0.04; 12 months F = 0.16, p = 0.69). There was no interaction between genotype and diet at 6 or 12 months. Individual comparisons were made using one-way ANOVA followed by Fisher`s post-hoc test (see fig. 1).

### GPR120 intestinal localization

**Figure 2:**
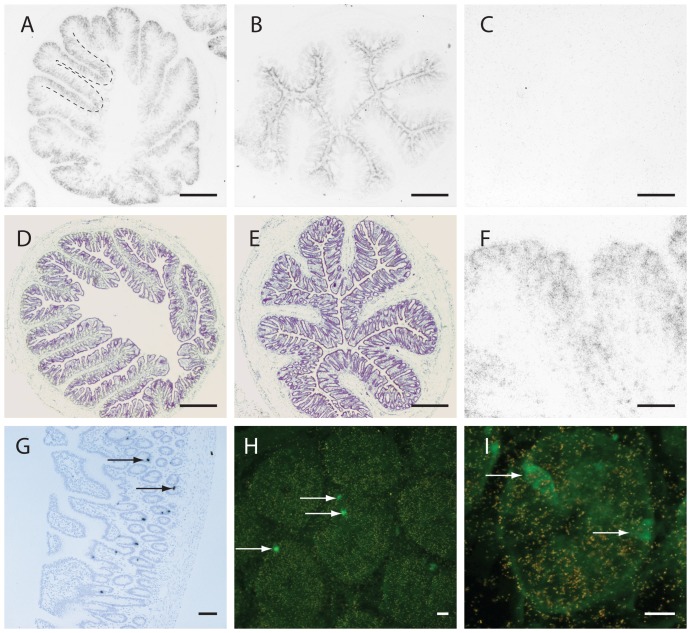
GPR120 and preproglucagon expression in the ileum and colon of the rat. Panel a and b: Low power photomicrographs of emulsion-dipped slides exposed to ileal (a) and colonic (b) sections hybridized with a ^33^P labelled cRNA antisense probe to rat GPR120. GPR120 expression is in virtually all epithelial cells lining the villi (villi in ileal section outlined with dashed line in (a)). Fig 2 f is a higher power microphotograph illustrating the expression of GPR120 (silver grains) in the epithelial cells in the ileum (f). No signal is seen in a section hybridized with a sense GPR120 cRNA (c). In contrast, to the widespread distribution of GPR120 mRNA preproglucagon is only expressed in the endocrine L-cell in the gut. Figure 2 g shows preproglucagon expression (two cells indicated by arrows) in the ileum of a rat (longitudinal section) (g). Figure 2 h and 2 i are high power fluorescence photomicrographs of ileal sections subjected to a double in situ hybridization procedure. Preproglucagon expressing neurons appear green (arrows) and GPR120 expressing cells have overlaying silver grains (h & i). Both preproglucagon and non-preproglucagon cells contain silvergrains showing that GPR120 expression is not restricted to the L-cell. Scale bars: a, b, d, e = 500 µm, c-f = 100 µm, g = 100 µm, h-i = 10 µm.

Examination of GPR120 mRNA localization in the terminal ileum and proximal colon of rat revealed that virtually all epithelial cells lining the villi express GPR120 (fig. 2a, b, d, e and f). In contrast to the widespread GPR120 expression, preproglucagon expression was limited to the endocrine L-cells in the gut (fig. 2 g). Both preproglucagon expressing cells and non-preproglucagon expressing cells showed GPR120 expression (fig. 2h and i).

### Oral linolenic acid tolerance test (OLTT)

**Figure 3:**
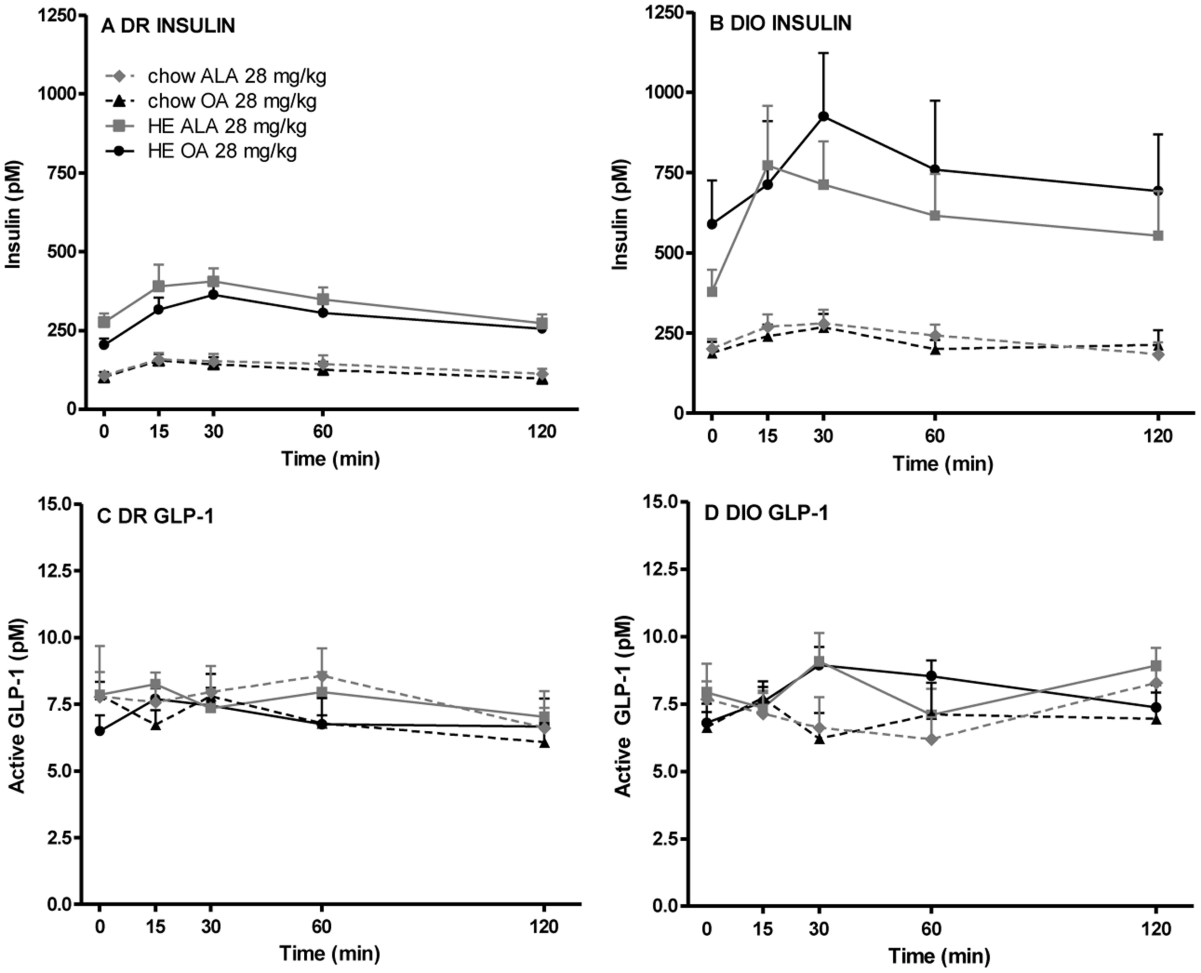
Plasma levels of insulin and active GLP-1 upon gavage with ALA or OA in DIO and DR rats. DIO and DR rats fed either chow or a HE diet received an acute gavage of 28/kg ALA or OA. N = 8. Both ALA and OA gavage caused a modest but non-significant increase in plasma insulin levels in all four groups. Two-way ANOVA with Bonferroni’s multiple comparisons correction (repeated measures) revealed no differences between the effect of ALA and OA in any of the four different rat groups. The statistical analysis revealed that GLP-1 plasma levels were not affected by either ALA or OA and did not differ between the four different rat groups (p<0.05 considered statistically significant). ALA: acid alpha-linolenic acid; OA: octanoic acid.

The acute effect of oral administration of ALA on plasma levels of insulin and active GLP-1 was first examined in DIO and DR rats fed either chow- or HE diet. Administration of either ALA or the control compound octanoic acid (OA) caused a modest but statistically insignificant increase in plasma insulin levels. However, two-way ANOVA with Bonferroni’s multiple comparisons correction (repeated measures) revealed no differences between the effect of ALA and OA on plasma insulin levels in any of the four different rat groups (fig. 3 a and b). Similarly, neither ALA nor OA administration affected the plasma levels of active GLP-1 in any of the rats (fig. 3 c and d).

**Figure 4:**
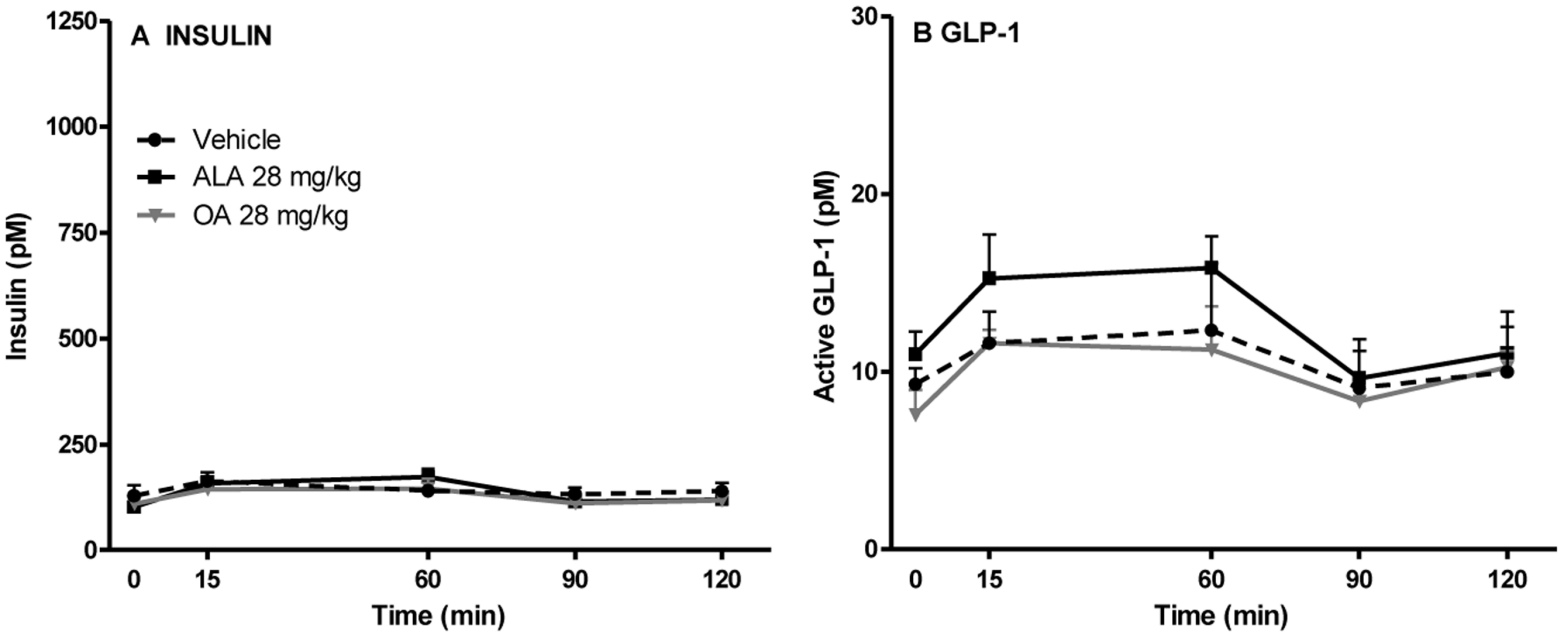
Plasma levels of insulin (a) and active GLP-1 (b) upon gavage with ALA or OA in Sprague-Dawley rats. Sprague-Dawley rats were gavaged with vehicle or 28 mg/kg ALA/OA, and the levels of active GLP-1 and insulin were determined. N = 6. Two-way ANOVA with Bonferroni’s multiple comparisons correction (repeated measures) revealed no differences between GLP-1 or insulin plasma levels after administration of ALA when compared both to OA and vehicle treated animals. ALA: acid alpha-linolenic acid; OA: octanoic acid.

Next, to also assess the effect of ALA in lean control rats, the acute effect on the plasma levels of active GLP-1 and insulin was determined in SPD rats following oral administration of ALA, OA or vehicle. Again no statistically significant differences were observed between the plasma levels of insulin or active GLP-1 levels in any of the groups (fig. 4 a and b).

**Figure 5:**
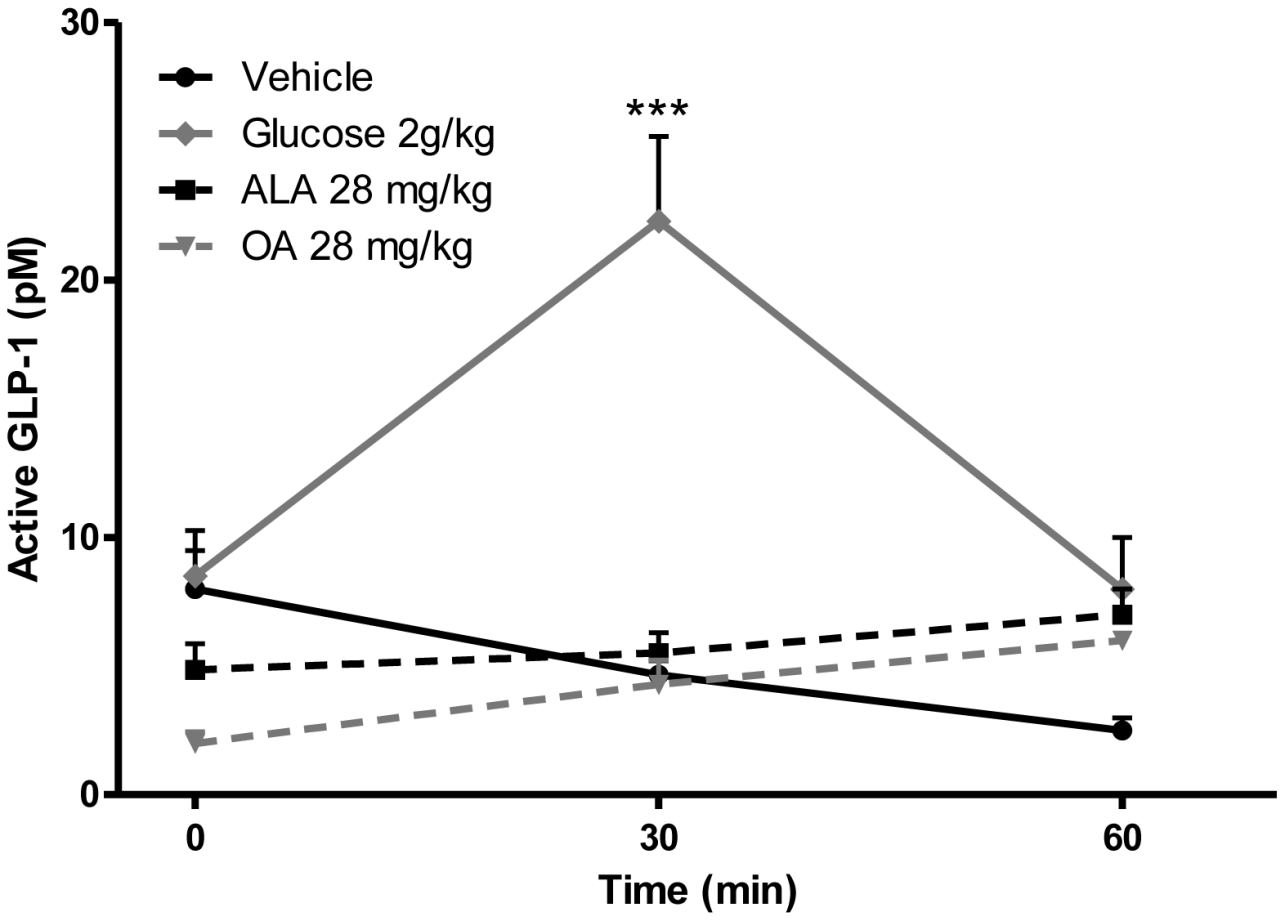
Plasma levels of total GLP-1 upon oral gavage with glucose, ALA or OA. Wistar rats were gavaged with vehicle, 2 g/kg glucose or 28 mg/kg ALA/OA. N = 10. No GLP-1 secretagogue effect was seen upon ALA/OA gavage, whereas glucose gavage stimulated GLP-1 release and hence raised the levels of total GLP-1 (Two way ANOVA, Bonferroni posttest). ALA: acid alpha-linolenic acid; OA: octanoic acid. *** = p<0.001.

Lastly, to verify the experimental set up, GLP-1 levels were measured in Wistar rats after an acute oral administration ALA, OA or glucose. No GLP-1 secretagogue effect was observed upon ALA or OA gavage, whereas glucose gavage statistically significantly increased total GLP-1 levels 30 min. after compound administration (fig. 5).

## Discussion

In the current paper we have reinvestigated the putative role of GPR120 as a specific target on endocrine L-cells potentially involved in GLP-1 secretion. We found that GPR120 is upregulated in obesity susceptible DIO rats when compared to diet resistant DR rats, and that the expression is not affected by diet (low or high fat).

These expression data are in line with recent findings of higher GPR120 gene and protein expression in the intestinal epithelium of outbred DIO vs. DR rats [11], as well as with findings from human fat depots (subcutaneous and omental) where GPR120 expression was found to be higher in obese vs. lean individuals [6]. Our finding of a non-nutrient dependent regulation of GPR120 is consistent with a previous report also indicating that GPR120 expression is not regulated by nutrient load [12]. The expression differences between DIO and DR rats in the gut coupled with the published data indicating that GPR120 is expressed exclusively on endocrine L-cells [1] prompted us to investigate the localization of the GPR120 receptor in the distal gut where the density of L-cells is highest. While our data indicate that GPR120 is indeed located on endocrine preproglucagon expressing (GLP-1/2) cells, the vast majority of GPR120 transcripts were actually found in the epithelial cells (non-endocrine). It should be noted that in the present analysis we used a ^33^P labelled GPR120 riboprobe and fresh frozen tissue for the in situ hybridizations, whereas Hirasawa *et al* used a digoxigenin labelled GPR120 probe on paraffin embedded tissue [1]. A direct labelling of the probe with a radioactive tracer as used in our study increases specificity (no risk of cross-reactivity between an antibody and tissue epitopes) and sensitivity dramatically over non-radioactive techniques, and we therefore consider that differences in protocols can explain the finding of much more widespread GPR120 expression in the gut than that reported by Hirasawa *et al* [1]. The present findings are in agreement with Sykaras *et al* who reported GPR120 to be expressed in both duodenal I- and non I-cells [13].

In the next series of experiments we tried to characterize effects of the GPR120 ligand ALA on GLP-1 release. In DIO and DR rats we did not observe baseline differences in GLP-1 plasma levels. Neither did we see any changes in GLP-1 plasma levels following an acute oral lipid tolerance test (OLTT) with the ω-3-FA and GPR120 agonist ALA. In order to exclude the possibility that DIO and DR rat responses to lipids were different from lean control rats, we performed additional experiments in two different strain (SPD and Wistar rats). In the last verification experiment we included a group of animals that were gavaged with glucose in order to demonstrate a GLP-1 response. Although we observed a clear effect of glucose on GLP-1 secretion we failed to see any effects of ALA on this L-cell derived hormone. This is in contrast to the initial observation from Hirasawa and co-workers examining the effect of ALA vs. vehicle on GLP-1 secretion in mice where they detected increases in GLP-1 and insulin levels in both portal vein as well as in inferior vena cava sampled blood after ALA administration [1]. Notably, in the present study we administered ALA in the same concentration as Hirasawa *et al* [1], so this parameter should not cause the observed differences. It should also be noted that in a follow up study in rats, Tanaka and co-workers found that co-administration of ALA and glucose increased plasma levels of GLP-1[14]. In our study, we did not co-administer ALA with glucose, but rather dosed ALA alone in order to determine direct effects of GPR120 receptor stimulation on GLP-1 release. It is possible that co-administration of ALA with glucose and/or a DPP-IV inhibitor can reveal slight effects of GPR120 stimulation on GLP-1 release. Nevertheless, the combination of our in vivo data and our in situ hybridization data showing widespread GPR120 expression in the gut epithelium indicate the role of GPR120 in the secretion of GLP-1 to be, at best, minor rather than major.

Collectively, the present data indicate that GPR120 does not play a major role in the regulation of GLP-1 secretion from intestinal L cells, and questions the notion that GPR120-mediated GLP-1 secretion induced by dietary free fatty acids might be important in the treatment of diabetes/obesity. However, the data also indicates that GPR120 is located in the gut epithelium in an excellent position to act as a sensor/and or transporter of ω-3-FAs. In light of the recent data on increased GPR120 expression in obese vs. lean humans, the widespread expression of GPR120 in the rat gut and the different expression levels in DIO vs. DR rats further supports a potential role for GPR120 in obesity susceptibility. Naturally, the above hypothesis should be further investigated.
